# Systematic Identification of the GH28 Gene Family in the Pathogenic Fungus *Cytospora pyri* and Functional Verification of the Candidate Virulence Gene *VP1G_08835*

**DOI:** 10.3390/jof12080569

**Published:** 2026-08-01

**Authors:** Ziyao Xue, Shasha Peng, Zhenzhen Liu, Yiwu Duan, Chengcai Yan, Lan Wang, Zhe Wang

**Affiliations:** 1 College of Agriculture, Tarim University, Alar 843300, China; 1031223212@stumail.taru.edu.cn (Z.X.); 107572024401@stumail.taru.edu.cn (S.P.); duanyiwu913@gmail.com (Y.D.); yancc119@126.com (C.Y.); 119920016@taru.edu.cn (L.W.); 2 Key Laboratory of Integrated Pest Management on Crops in Southern Xinjiang, Xinjiang Production and Construction Corps, Tarim University, Alar 843300, China; 3 College of Life Science and Technology, Tarim University, Alar 843300, China; 4 College of Information Engineering, Tarim University, Alar 843300, China; liu20210102@163.com

**Keywords:** pear Valsa canker, *Cytospora pyri*, GH28 gene family, gene deletion

## Abstract

Fragrant pear canker, caused by *Cytospora pyri*, is a major branch disease that reduces the productivity and longevity of Korla fragrant pear orchards in Xinjiang, China. During infection and lesion expansion in woody tissues, *C. pyri* must breach the host cell wall barrier, in which pectin degradation plays a central role by weakening intercellular adhesion and disrupting tissue integrity. Members of glycoside hydrolase family 28 (GH28), represented mainly by polygalacturonases and other pectin-degrading enzymes, are closely involved in fungal invasion and colonization. However, the composition and virulence-related functions of this gene family encoding for these enzymes in *C. pyri* remain unclear. In this study, GH28 genes were systematically identified and comparatively analyzed in *C. pyri* and closely related *Cytospora* species. Infection-stage expression profiling and functional validation were then performed to assess their roles in pathogenicity. A total of 73 GH28 genes were identified across five *Cytospora* species, including 15 in *C. pyri*. Most *C. pyri* GH28 proteins were predicted to be acidic, hydrophilic, extracellularly secreted proteins carrying conserved motifs. Phylogenetic analysis showed that *C. pyri* GH28 members were closely related to homologs from *C. mali*. Genomic distribution analysis revealed that these genes were dispersed across multiple scaffolds, with no obvious tandem duplication events. RT-qPCR analysis showed that all seven candidate GH28 genes were induced during infection of fragrant pear branches, with *VP1G_08835* and *VP1G_03209* exhibiting strong expression responses at 6 dpi. Functional validation further showed that deletion of *VP1G_08835* impaired vegetative growth and reduced lesion length on detached pear branches by 26.62% compared with the wild type, whereas complementation restored these phenotypes. These findings demonstrate that GH28 genes participate in *C. pyri* infection and identify *VP1G_08835* as an important GH28 member required for normal growth and contributing to virulence.

## 1. Introduction

Korla fragrant pear (*Pyrus sinkiangensis*) is an economically important and distinctive fruit crop in China, playing a vital role in the fruit industry of Xinjiang and in regional agricultural economic development. In recent years, pear canker caused by the fungal pathogen *Cytospora pyri* has become widespread in major pear-growing areas. A field survey conducted in Korla, Xinjiang, in 2018 reported disease incidence ranging from 44.00% to 84.44% [1]. The disease causes branch and trunk necrosis, reduced tree vigor, and even tree mortality, thereby posing a serious threat to the sustainable production of pear orchards. Therefore, elucidating the pathogenicity-related genes of *C. pyri* and their regulatory mechanisms may improve our understanding of disease development and provide a molecular basis for future disease-control research [2].

The plant cell wall represents the first physical barrier that pathogenic fungi must overcome during host infection, with pectin serving as a major component of the middle lamella and primary cell wall. To breach this barrier, pathogenic fungi secrete a wide range of cell wall-degrading enzymes that remodel host cell wall architecture, thereby facilitating penetration, colonization, and lesion expansion. Glycoside hydrolase family 28 (GH28) primarily comprises pectin-degrading enzymes, including polygalacturonases, which hydrolyze α-1,4-glycosidic bonds in the pectin backbone and play important roles in fungal pathogenesis [3]. Unlike general degradative enzymes, GH28 family members have dual biological significance in pathogen–host interactions. On the one hand, their enzymatic activity directly compromises host cell wall integrity, creating favorable conditions for pathogen invasion and lesion development. On the other hand, pectin-derived fragments may be perceived by the host as damage-associated molecular patterns [4,5], thereby activating defense responses. In addition, plants can restrict pathogen-derived polygalacturonase activity through defense-related proteins, such as polygalacturonase-inhibiting proteins (PGIPs) [6]. Thus, the GH28 enzyme system not only functions as a cell wall-degrading machinery during infection but also represents a key component linking pathogen virulence and host immune recognition. Previous studies have shown that GH28 members in phytopathogenic fungi, such as the necrotrophic fungus *Botrytis cinerea* and *Fusarium* spp., are involved not only in host cell wall degradation but also in virulence and host adaptation [7]. For example, several endopolygalacturonase genes in *B. cinerea* show host-dependent expression patterns [8] during infection, and deletion of Bcpg1 significantly reduces the pathogen’s capacity to colonize and expand within various host tissues [9]. Moreover, GH28 polygalacturonases secreted by *Fusarium* species can be recognized or inhibited by plant defense-related proteins, further highlighting the biological importance of this enzyme family in pathogen–host interactions [6,10]. However, the roles and relative contributions of individual GH28 proteins may differ among pathogen–host systems. Therefore, the examples above should be interpreted as evidence for the context-dependent functions of GH28 proteins rather than as support for a universal mechanism.

Although the GH28 gene family has been extensively studied in several model phytopathogenic fungi, the member composition, structural characteristics, evolutionary relationships, and infection-related roles of this family remain poorly understood in *C. pyri*, the causal agent of Korla fragrant pear canker. In particular, it remains unclear whether *C. pyri* harbors GH28 candidate genes closely associated with pear branch infection and whether these genes contribute to fungal growth, colonization, and virulence. Identification of GH28 members based solely on genome annotation is insufficient to determine their functions during host infection. Therefore, in this study, we systematically identified and characterized GH28 gene family members in *C. pyri* and closely related *Cytospora* species. We further examined the expression patterns of candidate GH28 genes during infection of Korla fragrant pear branches using RT-qPCR and selected *VP1G_08835*, which exhibited a strong transcriptional response, for functional validation to determine its roles in the growth and pathogenicity of *C. pyri*. This study aimed to clarify the relationship between the GH28 enzyme system and the pathogenesis of Korla fragrant pear canker at both the gene family and single-gene functional levels, thereby providing a theoretical foundation for understanding the infection mechanisms of *C. pyri* and identifying potential targets for disease control.

## 2. Materials and Methods

### 2.1. Experimental Materials

*C. pyri* isolate KEL18 was used as the wild-type isolate in this study, and L48 was used as its laboratory working code. KEL18 and L48 refer to the same biological isolate. The isolate was originally recovered from bark tissues collected at the junction between necrotic and apparently healthy tissues of symptomatic Korla fragrant pear branches in Alar, Xinjiang, China. Its isolation, purification, morphological and molecular identification, and pathogenicity assessment were described in detail by Wang (2023) [11]. Its pathogenicity was confirmed by inoculating healthy pear tissues, followed by the re-isolation and identification of the inoculated fungus in accordance with Koch’s postulates.

The isolate is currently maintained in the culture collection of the Key Laboratory of Integrated Pest Management on Crops in Southern Xinjiang, Xinjiang Production and Construction Corps, Tarim University. Healthy, disease-free Korla fragrant pear branches of similar age and diameter used for the inoculation assays were collected from the Horticultural Experimental Station of Tarim University in Alar, Xinjiang, China. For routine cultivation, isolate KEL18 was grown on potato dextrose agar (PDA; 200 g peeled potatoes, 20 g glucose (Tianjin Xinbote Chemical Co., Ltd., Tianjin, China), 20 g agar powder (cat. no. A8190, Beijing Solarbio Science & Technology Co., Ltd., Beijing, China), and distilled water to a final volume of 1 L) at 25 °C in the dark in an SPX-series biochemical incubator (Ningbo Jiangnan Instrument Factory, Ningbo, China). For long-term preservation, mycelial plugs of the isolate were stored in 25% (v/v) glycerol (analytical grade; Fuchen (Tianjin) Chemical Reagent Co., Ltd., Tianjin, China) at −80 °C in an ultra-low-temperature freezer (MD-86L456K, Midea Biomedical Co., Ltd., Hefei, China).

### 2.2. Identification of GH28 Family Members

Whole-genome data for *C. pyri* (Assembly ID: ASM81338v1), *C. mali* (Assembly ID: ASM81815v1), *C. chrysosperma* (Assembly ID: ASM379527v1), *C. schulzeri* (Assembly ID: ASM379531v1), and *C. paraplurivora* (Assembly ID: ASM2127294v2) were downloaded from the NCBI database. The hidden Markov model (HMM) profile of the GH28 family, corresponding to protein domain PF00295, was obtained from the Pfam database [12]. HMMER v3.4 searches [13] were performed in TBtools v2.119 [14] using the GH28 HMM profile against the whole-genome protein sequences of the five species to identify candidate GH28 proteins. The candidate proteins were subsequently verified using the CDD database [15], and those containing a complete GH28 domain (PF00295) were retained as the final GH28 family members.

### 2.3. Protein Sequence Analysis and Structural Characterization

The physicochemical properties of *C. pyri* GH28 proteins, including amino acid length, molecular weight, theoretical isoelectric point (pI), instability index, aliphatic index, and grand average of hydropathicity (GRAVY), were analyzed using the ExPASy ProtParam [16] tool. Subcellular localization was predicted using CELLO v2.5 [17]. Conserved motifs were identified using MEME Suite, with the maximum number of motifs set to 10 and the maximum motif width set to 50 amino acids [18]. Gene structures, including exon–intron organization, were analyzed using TBtools based on genome annotation files. The conserved motif and gene structure results were subsequently integrated and visualized in TBtools.

The N-terminal signal peptide and its cleavage site of VP1G_08835 were predicted using SignalP 6.0 [19], and the targeting-peptide cleavage site was independently assessed using TargetP 2.0 [20]. Protein domains and domain architectures were annotated using InterPro [21] and Pfam. Intrinsically disordered regions were predicted using MobiDB-lite 3.0 [22], and transmembrane topology was analyzed using DeepTMHMM [23].

Three-dimensional structures of the 15 *C. pyri* GH28 proteins were predicted using the Phyre2 Protein Homology/analogY Recognition Engine v2.0 through batch processing [24]. The final structural model generated for each protein was downloaded in PDB format and used for subsequent structural analysis. The experimentally determined structure of the fungal GH28 endo-polygalacturonase TlPGA from *Talaromyces leycettanus* JCM 12802 (PDB ID: 6KVE, chain A) was obtained from the Protein Data Bank and used as the reference structure [25].

Structural superposition was performed in PyMOL (Version 3.1.0, Schrödinger, LLC, New York, NY, USA) using Cα atoms. The final post-refinement Cα-RMSD values and aligned Cα-atom counts obtained after iterative outlier rejection were recorded. Target-model coverage was calculated as the number of aligned Cα atoms divided by the total number of Cα atoms present in the corresponding Phyre2 final model. The 6KVE-A coverage was calculated as the number of aligned Cα atoms divided by the 339 Cα atoms present in the reference structure used for superposition. Coverage values were expressed as percentages.

### 2.4. Phylogenetic Analysis of the GH28 Gene Family

A total of 73 GH28 protein sequences were collected from five species, including *C. pyri*, *C. mali*, *C. chrysosperma*, *C. schulzeri*, and *C. paraplurivora*. Redundant sequences were removed using CD-HIT v4.8.1 [26] with a sequence identity threshold of 90%, resulting in 69 retained sequences. These sequences were further verified against the Pfam HMM profile, and only those containing a complete PF00295 domain were selected for subsequent analysis. Orthologous clustering was performed using OrthoFinder v2.5.4 [27] with an MCL inflation parameter of 1.5. For each gene cluster, the longest sequence or the sequence with the most complete annotation was selected as the representative sequence for phylogenetic tree construction.

A maximum likelihood (ML) phylogenetic tree was constructed using IQ-TREE 2 v2.2.0 [28]. The best-fit substitution model, LG + G, was determined using the built-in ModelFinder [29] module. Node support was assessed using 1000 ultrafast bootstrap replicates and 1000 SH-aLRT tests with the following command: iqtree2 -s example.phy -m LG + G -B 1000 -alrt 1000 -T 2. Only nodes with support values of ≥70% were displayed. The final consensus tree (.contree) was visualized using FigTree v1.4.4. In addition, an independent ML tree was constructed using MEGA 12 [30] with 1000 bootstrap replicates to verify the consistency of the phylogenetic results.

### 2.5. Analysis of Promoter Cis-Acting Elements

To predict cis-acting elements in the promoter regions of *C. pyri* GH28 genes, 2000 bp upstream sequences were extracted using TBtools based on the *C. pyri* genome sequence and annotation files. Cis-acting elements in these promoter sequences were predicted using the PlantCARE [31] database. Non-specific elements, such as TATA-box-derived elements without functional annotation, were removed, and elements with similar annotations were merged. Only elements with clearly defined biological functions were retained for subsequent analysis and visualization.

### 2.6. Sequence Distribution and Intraspecific Collinearity Analysis

Based on the *C. pyri* genome data, intraspecific collinearity analysis of GH28 gene family members was performed using TBtools with the MCScanX [32] algorithm to detect locally collinear blocks (LCBs) and potential tandem duplication or large-scale fragment rearrangement events. The distribution of GH28 genes on scaffolds was also analyzed to investigate the expansion patterns of this gene family.

### 2.7. Collection of Infection Samples and RT-qPCR Expression Analysis

The mycelial plug inoculation method was used to analyze the expression patterns of *C. pyri* GH28 candidate genes during infection of Korla fragrant pear branches. Mycelial plugs with a diameter of 5 mm were excised from the margins of actively growing 3 d *C. pyri* colonies and inoculated onto artificially wounded sites on surface-disinfected healthy pear branches. For the control treatment, sterile PDA plugs of the same diameter were placed onto wounded branches. After inoculation, the branches were placed in moist chambers lined with wet filter paper and incubated at 25 °C. Tissues from the margin between diseased and healthy areas were collected at 0 h, 12 h, 2 d, 4 d, 6 d, and 8 d post-inoculation, immediately frozen in liquid nitrogen, and stored at −80 °C until RNA extraction. For each time point, tissues were collected from three independently inoculated pear branches, which served as three biological replicates. RNA extraction and cDNA synthesis were performed independently for each biological replicate, and each cDNA sample was analyzed in three technical replicates by RT-qPCR.

Total RNA was extracted using the TRNzol Universal Total RNA Extraction Kit, and contaminating genomic DNA was removed. RT-qPCR was performed using the Universal SYBR Green Master Mix on a LightCycler 480 II Real-Time PCR System. The actin gene of *C. pyri* was used as the internal reference gene for expression normalization, following its previous application in RT-qPCR analyses of *Valsa pyri* [33]. Relative expression levels of the candidate GH28 genes were calculated using the 2^−ΔΔCt^ method [34]. All primers were designed using SnapGene, and the primer sequences are listed in Table 1. Amplification specificity was confirmed by melting curve analysis. Statistical significance was assessed using one-way ANOVA followed by Tukey’s multiple comparison test.

### 2.8. Generation of Gene Deletion Mutants, Complemented Strains, and Overexpression Strains

To generate deletion mutants, a gene knockout cassette was constructed using double-joint PCR [35]. Approximately 1500 bp upstream and downstream flanking regions of *VP1G_08835* were fused with the G418 resistance marker to generate the knockout cassette. The cassette was introduced into *C. pyri* protoplasts by PEG-mediated transformation [33]. Transformants were selected on TB_3_ medium containing 100 μg·mL^−1^ G418, and target gene deletion mutants were identified by PCR using four pairs of primers (Supplementary Figure S2A).

To obtain complemented strains, a fragment containing the full-length *VP1G_08835* gene and its 1.5 kb native promoter region was amplified using the CF/R primer pair and cloned into the PDL2 vector by gap-repair cloning. The resulting construct was introduced into protoplasts of the deletion mutant strain. Transformants were selected on medium containing 100 μg·mL^−1^ hygromycin, and complemented strains were identified by PCR using the *VP1G_08835*-1F/4R primer pair. The primers used in this study are listed in Supplementary Table S1.

To construct overexpression strains, the complete coding sequence of *VP1G_08835* without its native promoter and introns was amplified. The PCR product was cloned into the PDL2 vector using the ClonExpress II One Step Cloning Kit (Vazyme, Nanjing, China). After sequence verification, the recombinant plasmid was introduced into wild-type *C. pyri* protoplasts. Transformants were selected on TB3 plates containing 100 μg·mL^−1^ hygromycin, and stable overexpression strains were obtained after confirmation of *VP1G_08835* copy number by qPCR and transcript levels by RT-qPCR.

### 2.9. Colony Growth and Virulence Assays on Detached Branches

The wild-type strain, Δ*VP1G_08835* mutant, complemented strain, and overexpression strains were inoculated at the center of PDA plates and incubated at 25 °C in the dark for 3 d. Colony diameters were measured daily, and three biological replicates were included for each strain. Healthy, disease-free, one-year-old Korla fragrant pear branches of similar diameter were cut into 20 cm-long segments. The branch segments were surface-disinfested with 3% (*v*/*v*) sodium hypochlorite for 10 min, thoroughly rinsed with sterile distilled water, and air-dried. Both ends of each segment were sealed with paraffin to minimize water loss. One wound was made on each branch segment using a sterile 5 mm cork borer, and the bark tissue was removed to expose the xylem. A 5 mm mycelial plug was placed onto each wound with the mycelial side facing the exposed tissue. Sterile PDA plugs of the same size were used as negative controls. After inoculation, the branches were placed in moist chambers and incubated at 25 °C for 5 d, after which lesion lengths were measured. At least three biological replicates were included for each strain.

### 2.10. Statistical Analysis

Data were analyzed using one-way analysis of variance (ANOVA) followed by Tukey’s post hoc test, with the significance threshold set at *p* < 0.05. All statistical analyses were performed using IBM SPSS Statistics version 20.0 (IBM Corp., Armonk, NY, USA), and graphs were generated using GraphPad Prism 9 (GraphPad Software, Inc., San Diego, CA, USA). Lesion lengths were measured using ImageJ 1.53 software (National Institutes of Health, Bethesda, MD, USA) [36] software.

## 3. Results

### 3.1. Identification and Physicochemical Characterization of the GH28 Gene Family in Cytospora pyri

A total of 73 GH28 family members were identified from five *Cytospora* species, including 15 in *C. pyri*, 16 in *C. mali*, 15 in *C. chrysosperma*, 14 in *C. schulzeri*, and 13 in *C. paraplurivora*. The amino acid lengths of the *C. pyri* GH28 proteins ranged from 327 to 758 aa, with most proteins concentrated between 300 and 500 aa. Their molecular weights varied from 35,573.51 to 82,574.07 Da, and their theoretical isoelectric point (pI) values ranged from 4.11 to 5.22, indicating that these proteins are generally acidic. This acidic property may be associated with their solubility and stability in acidic microenvironments, potentially reflecting adaptation to the localized acidification of host cell walls during infection [37]. The instability indices ranged from 23.61 to 44.12, with values below 40 indicating relatively stable proteins, whereas the aliphatic indices ranged from 67.83 to 88.46. GRAVY values varied from −0.596 to 0.087. Except for *VP1G_08494* (0.087) and *VP1G_03614* (0.003), which showed slightly positive values, the remaining 13 members had negative GRAVY values, suggesting that most *C. pyri* GH28 proteins are hydrophilic. This feature is consistent with their putative roles as secreted cell wall-degrading enzymes functioning in extracellular aqueous environments [3]. Subcellular localization analysis showed that *VP1G_03071* was predicted to localize to the cytoplasm, whereas the other 14 members were predicted to be extracellular secretory proteins (Table 1).

### 3.2. Conserved Motif and Gene Structure Analyses

**Figure 1:**
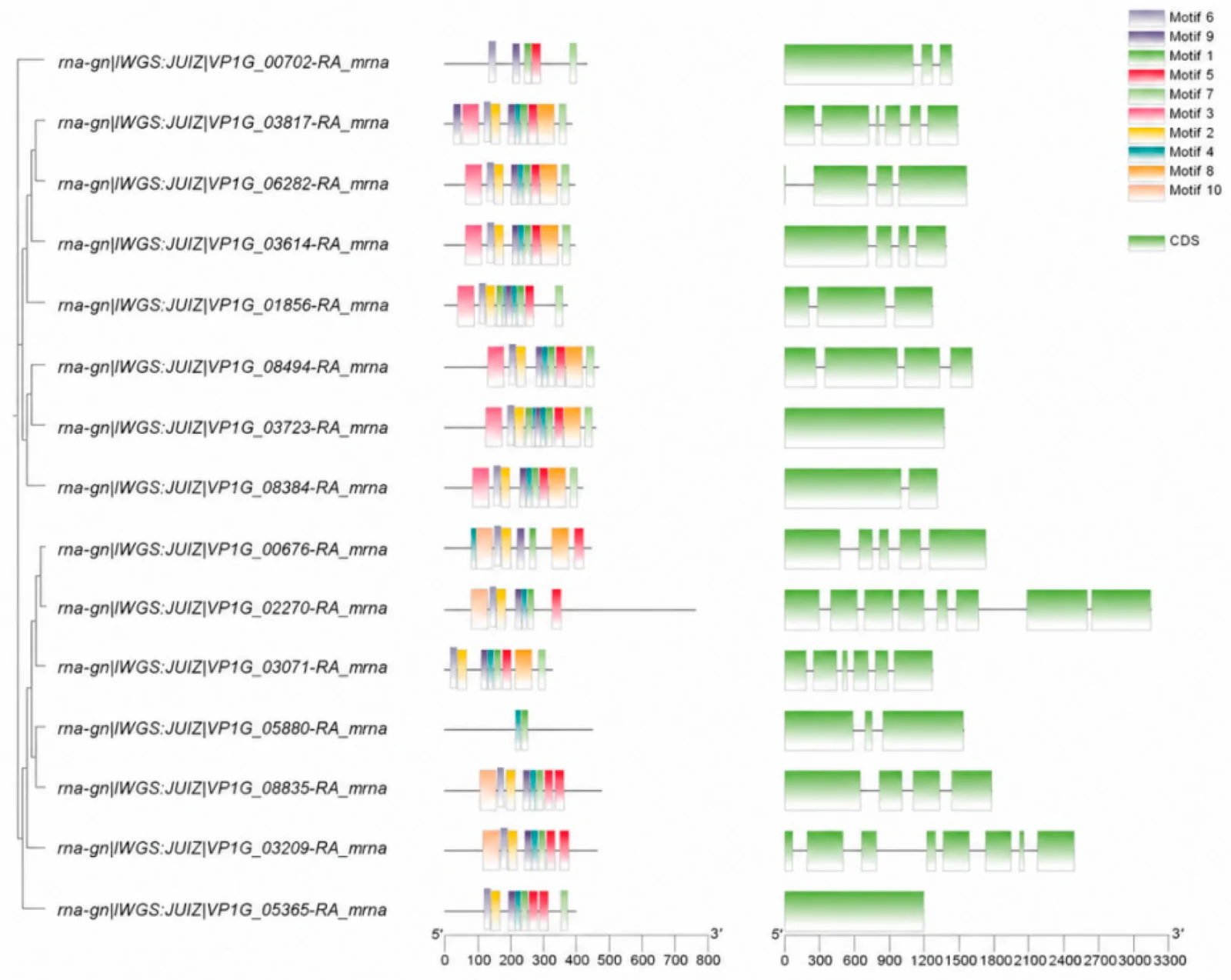
Phylogenetic relationships, conserved motifs, and gene structures of *C. pyri* GH28 genes.

A total of 10 conserved motifs were identified by MEME analysis (Figure 1). Motifs 1, 4, and 5 were highly conserved in most GH28 members. Their distribution overlaps with conserved regions characteristic of fungal GH28 proteins and the parallel β-helical fold reported for structurally characterized GH28 polygalacturonases [3,38]. In contrast, *VP1G_05880* contained only two motifs, whereas *VP1G_03817* and *VP1G_03723* contained all 10 motifs, suggesting structural divergence among family members. Gene structure analysis revealed substantial differences in exon–intron number and organization. *VP1G_03723* and *VP1G_05365* exhibited single-exon, intronless structures, whereas both *VP1G_03209* and *VP1G_02270* contained eight exons and seven introns. This structural diversity may be closely associated with functional differentiation among GH28 family members.

### 3.3. Structural Comparison of Phyre2-Predicted C. pyri GH28 Protein Models with 6KVE-A

To assess structural conservation within the *C. pyri* GH28 family, the Phyre2-predicted structural models of all 15 GH28 proteins were superimposed onto the experimentally determined GH28 endo-polygalacturonase structure 6KVE-A using PyMOL [25]. All 15 models contained regions that could be structurally aligned with the reference structure. The post-refinement Cα-RMSD values ranged from 0.416 to 1.333 Å, with 136–296 Cα atoms retained in the final structural alignments. Target-model coverage ranged from 36.8% to 85.8%, whereas coverage of the 6KVE-A reference structure ranged from 40.1% to 87.3% (Table 2).

Seven proteins—VP1G_01856, VP1G_03614, VP1G_03723, VP1G_03817, VP1G_06282, VP1G_08384, and VP1G_08494—showed relatively extensive structural similarity to 6KVE-A. Their post-refinement Cα-RMSD values ranged from 0.416 to 0.637 Å, with 245–296 aligned Cα atoms. Target-model coverage ranged from 71.8% to 85.8%, and 6KVE-A coverage ranged from 72.3% to 87.3%. These results support broad predicted conservation of the canonical GH28 fold in these protein models.

The remaining models showed lower structural coverage, although their aligned regions generally exhibited relatively low Cα-RMSD values. This pattern suggests that these proteins retain a conserved GH28 structural region, whereas other portions of the models may be structurally divergent or may contain variable terminal extensions, insertions, or loop regions.

The VP1G_08835 model contained 163 Cα atoms aligned with 6KVE-A and had a post-refinement Cα-RMSD of 0.912 Å. Its target-model coverage and 6KVE-A coverage were 41.9% and 48.1%, respectively. Therefore, VP1G_08835 showed partial or domain-level structural conservation with 6KVE-A rather than extensive global structural similarity.

### 3.4. Phylogenetic Analysis

**Figure 2:**
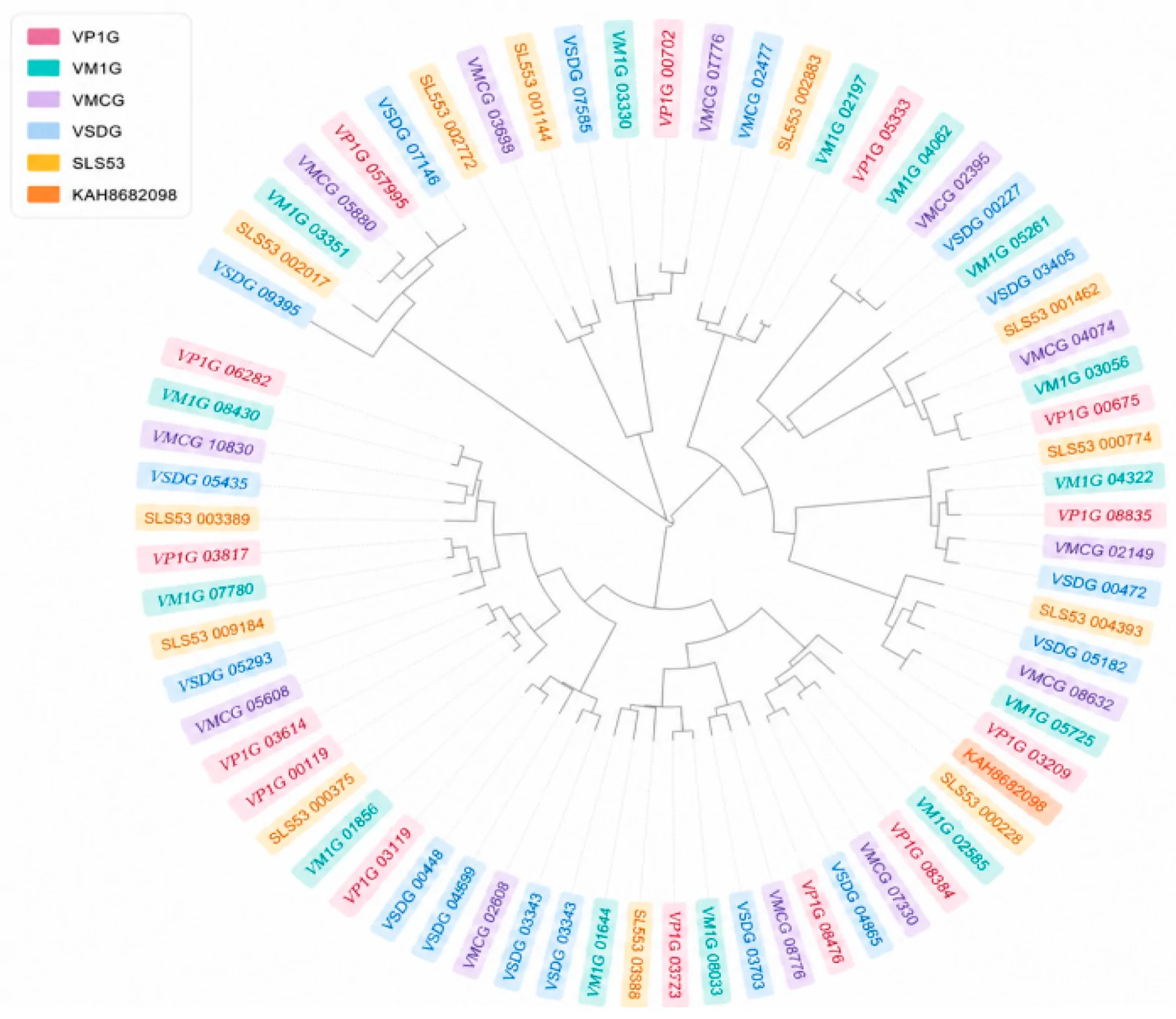
Phylogenetic tree of the GH28 gene family in five *Cytospora* species.

The ML phylogenetic tree constructed using 69 non-redundant GH28 protein sequences revealed the evolutionary relationships of the GH28 family within the genus *Cytospora* (Figure 2). In terms of tree topology, homologous genes from *C. pyri* and *C. mali*, represented by the VP1G and VM1G prefixes, respectively, frequently clustered as sister pairs, such as *VP1G_06282/VM1G_08430* and *VP1G_03817/VM1G_07780*, with relatively short branch lengths. This pattern indicates a close evolutionary relationship between these two species and suggests that many GH28 family members have retained strong orthologous relationships. Similarly, genes from *C. chrysosperma* and *C. schulzeri*, represented by the VSDG and VMCG prefixes, respectively, were frequently clustered adjacent to each other, suggesting a close evolutionary relationship between these species. In contrast, genes from *C. paraplurivora*, represented by the SLS53 prefix, were widely distributed across the major branches of the tree but were generally positioned outside the VP1G/VM1G and VSDG/VMCG pairs, indicating that *C. paraplurivora* diverged earlier than the other four species.

Differences in branch length indicated substantial heterogeneity in evolutionary rates within the GH28 family. The branch containing *VSDG_09395* was exceptionally long, suggesting that this gene may have undergone rapid sequence evolution or functional specialization and may represent a functionally diverged paralog. The GH28 family showed evidence of expansion in the examined species and formed multiple evolutionary subfamilies, with major gene duplication events likely occurring before species divergence [38]. This expansion pattern may reflect the functional diversification of GH28 enzymes required for pathogen adaptation to different hosts or for the utilization of diverse pectin substrates.

### 3.5. Analysis of Promoter Cis-Acting Elements

**Figure 3:**
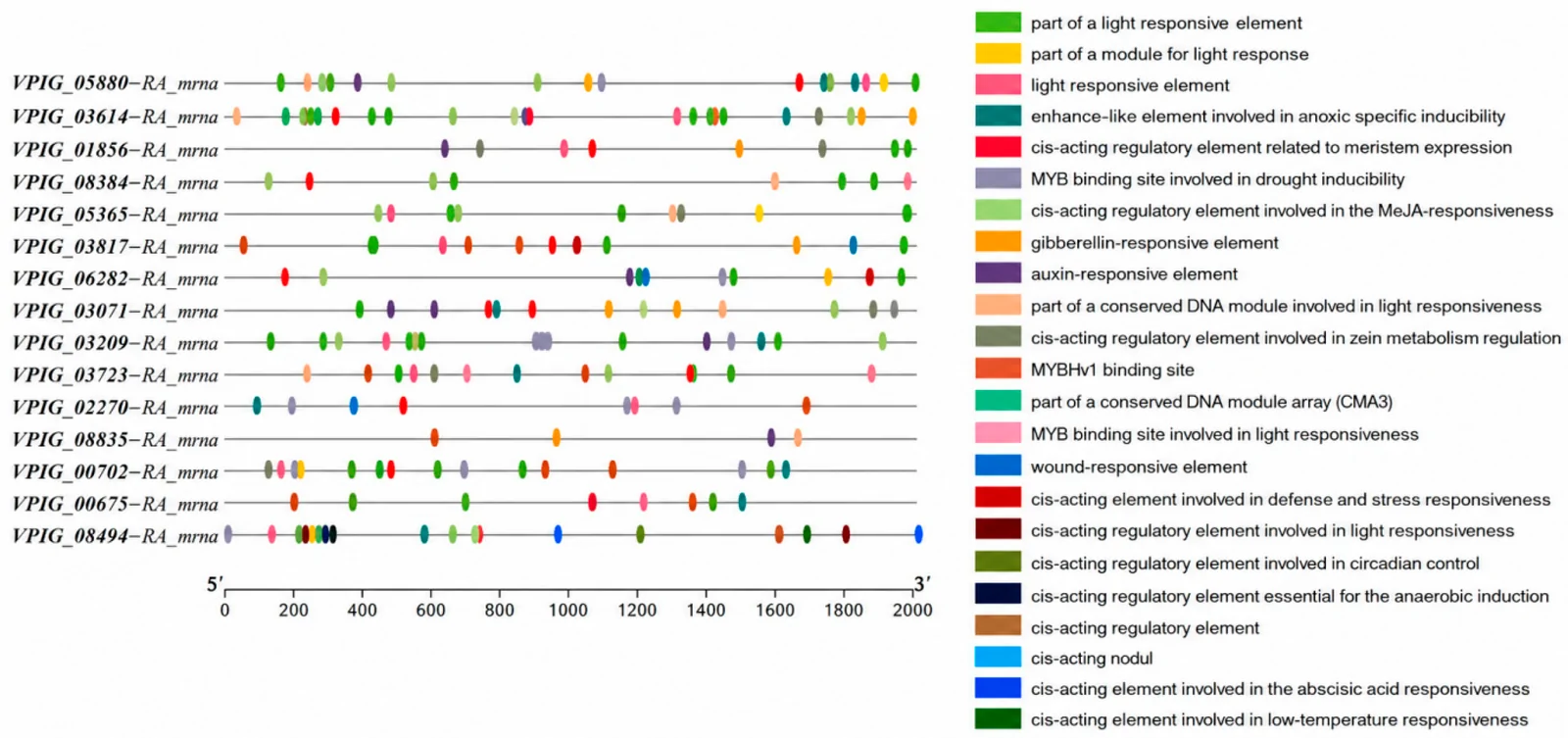
Distribution of cis-acting elements in the promoters of *C. pyri* GH28 genes.

Cis-acting elements were predicted in the 2000 bp upstream regions relative to the start codons of *C. pyri* GH28 genes (Figure 3). Several categories of regulatory elements were identified, including hormone-responsive elements associated with gibberellin, auxin, abscisic acid (ABA), and methyl jasmonate (MeJA) responses, as well as light-responsive, low-temperature-responsive, defense/stress-responsive, and wound-responsive elements. The presence of these elements suggests that the expression of *C. pyri* GH28 genes may be coordinately regulated by multiple external signals, allowing flexible modulation of enzymatic activity during infection in response to host defense status and environmental conditions. The presence of low-temperature-responsive elements may also be associated with the overwintering adaptation of this pathogen in Xinjiang.

### 3.6. Genomic Distribution and Intraspecific Collinearity Analysis of GH28 Genes

**Figure 4:**
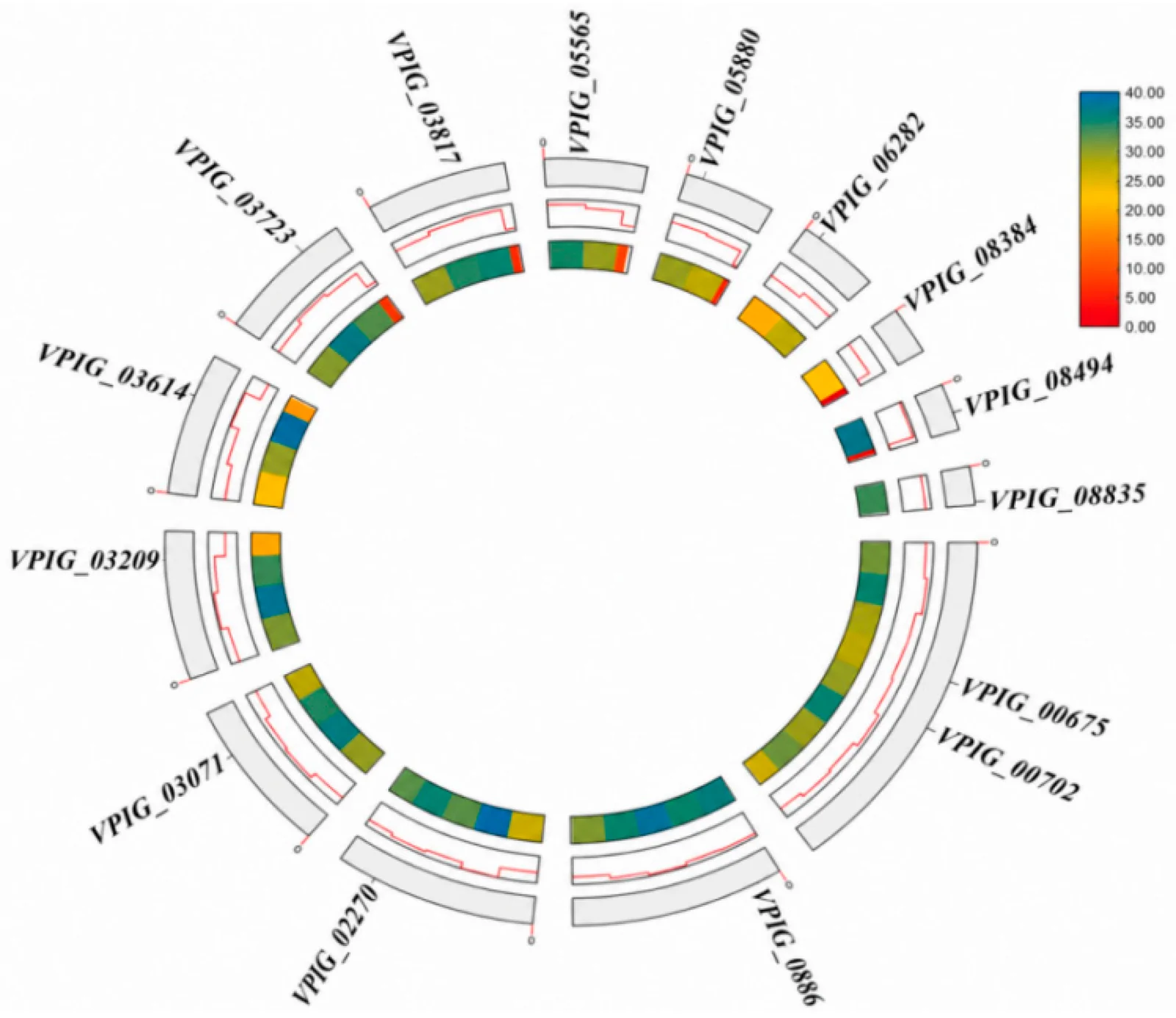
Distribution of *C. pyri* GH28 genes and inter-scaffold collinearity relationships.

Intraspecific collinearity analysis performed using TBtools with the MCScanX algorithm showed that the 15 GH28 genes of *C. pyri* were dispersed across 14 scaffolds (Figure 4). Only one scaffold contained two GH28 genes, whereas each of the remaining scaffolds contained a single GH28 gene. No obvious positive correlation was observed between scaffold length and GH28 gene number. Collinearity analysis did not detect any collinear blocks among GH28 genes, indicating that tandem duplication may not have contributed substantially to the expansion of the GH28 gene family in *C. pyri*. Instead, this family may have expanded mainly through dispersed duplication mechanisms, which may help reduce functional redundancy and promote the differentiation and diversification of substrate specificity among family members.

### 3.7. Expression Analysis of GH28 Genes During Infection of Korla Fragrant Pear Branches

**Figure 5:**
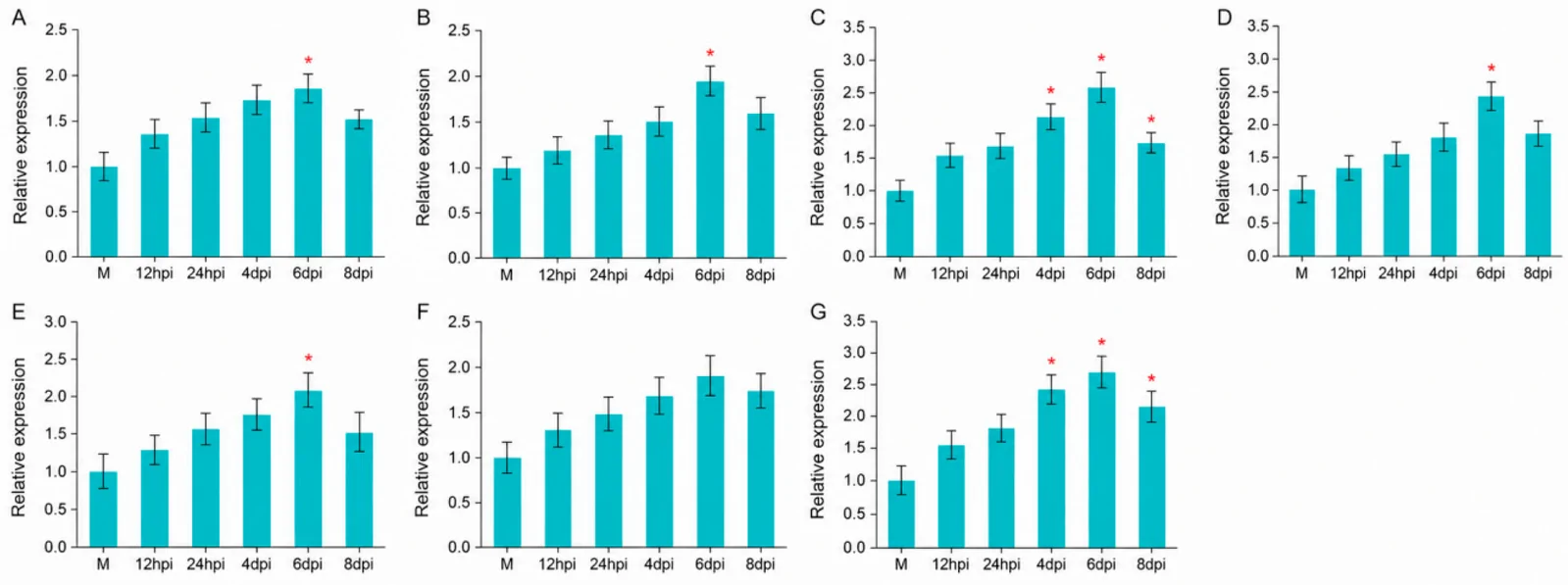
Relative expression levels of *C. pyri* GH28 candidate genes at different time points during infection of Korla fragrant pear branches. (**A**) *VP1G_00675*; (**B**) *VP1G_03071*; (**C**) *VP1G_03209*; (**D**) *VP1G_05365*; (**E**) *VP1G_05880*; (**F**) *VP1G_08384*; and (**G**) *VP1G_08835*. Asterisks indicate significant differences: *, *p* < 0.05.

To further identify GH28 candidate genes associated with *C. pyri* infection, seven GH28 genes were selected based on their predicted relevance to pathogenicity, and their expression patterns at different time points during infection of Korla fragrant pear branches were examined by RT-qPCR (Figure 5). The seven candidate GH28 genes showed diverse induction patterns after inoculation suggesting that these GH28 members may be involved in the infection of Korla fragrant pear branches by *C. pyri* [8].

Based on the intensity of transcriptional responses and the timing of peak expression, the tested genes were classified into three groups. The first group comprised strongly responsive genes, including *VP1G_08835* and *VP1G_03209*. *VP1G_08835* showed a continuous increase in expression after inoculation and reached its highest expression level at 6 days post-inoculation (dpi), with an expression level approximately 2.77-fold higher than that of the control. This gene exhibited the strongest response among all tested genes. *VP1G_03209* was also significantly upregulated during infection and peaked at 6 dpi, with an expression level approximately 2.57-fold higher than that of the control. These results indicate that *VP1G_08835* and *VP1G_03209* may represent important candidate genes closely associated with the infection process among the GH28 family members of *C. pyri*. The second group consisted of moderately responsive genes, including *VP1G_05365* and *VP1G_05880*. These two genes were continuously upregulated after inoculation, with peak expression also occurring around 6 dpi, reaching approximately 2.44- and 2.15-fold higher levels than those of the control, respectively. This expression pattern suggests that they may be involved in host cell wall degradation or lesion expansion during the middle to late stages of infection. The third group included mildly responsive genes, namely *VP1G_00675*, *VP1G_03071*, and *VP1G_08384*. These genes also showed increased expression after inoculation, but the magnitude of induction was relatively low, suggesting that they may play auxiliary roles during infection.

Among the seven GH28 genes examined, *VP1G_08835* showed the strongest and most sustained infection-induced expression, reaching approximately 2.77-fold at 6 dpi. It was also predicted to encode an extracellular protein containing a complete GH28 domain. Structural comparison further showed that VP1G_08835 retained a partial GH28 structural region similar to 6KVE-A, with a Cα-RMSD of 0.912 Å, a target-model coverage of 41.9%, and a reference-structure coverage of 48.1%. Because its structural coverage was not the highest among the family members, the structural result was regarded as complementary rather than decisive evidence. *VP1G_08835* was therefore selected for functional validation primarily because of its strong infection-associated expression and predicted extracellular GH28 characteristics.

### 3.8. VP1G_08835 Contributes to Fungal Growth and Virulence in C. pyri

**Figure 6:**
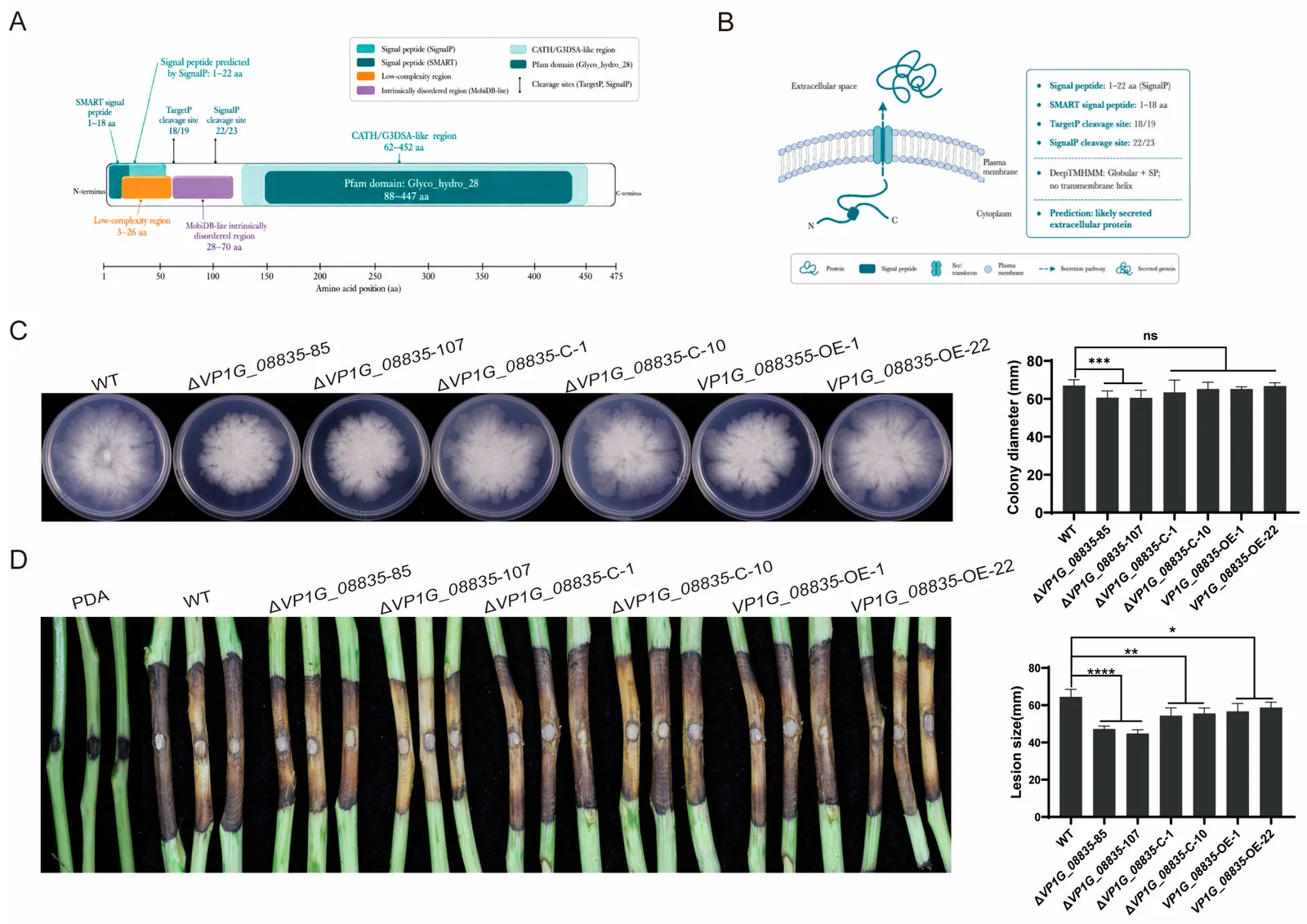
Structural prediction of *VP1G_08835* and phenotypic analysis of the related strains. (**A**) Domain organization and signal peptide prediction of the *VP1G_08835*-encoded protein. (**B**) Prediction of the secretion characteristics and subcellular localization of *VP1G_08835*. (**C**) Colony morphology and statistical analysis of colony diameter for different strains grown on PDA. (**D**) Lesion phenotypes and lesion length measurements on detached pear twigs inoculated with different strains.Data are presented as mean ± SD (n = 3). Asterisks indicate significant differences (* *p* < 0.05; ** *p* < 0.01; *** *p* < 0.001; **** *p* < 0.0001), and “ns” indicates no significant difference (*p* ≥ 0.05), as determined by Student’s *t*-test (or one-way ANOVA with Tukey’s post-hoc test).

To preliminarily characterize the structural features of the protein encoded by *VP1G_08835*, its amino acid sequence was subjected to bioinformatic analysis. The results showed that the protein contains an N-terminal signal peptide with a predicted cleavage site. The central region harbors a conserved domain and also contains low-complexity and intrinsically disordered regions (Figure 6A). Further analysis indicated that the *VP1G_08835* protein lacks obvious transmembrane helices and is likely secreted extracellularly via the signal peptide-mediated secretory pathway (Figure 6B).

To investigate the function of *VP1G_08835* in *C. pyri*, the gene was deleted using double-joint PCR and polyethylene glycol (PEG)-mediated protoplast transformation. Two deletion mutants, Δ*VP1G_08835-85* and Δ*VP1G_08835-107*, were obtained. The full-length *VP1G_08835* sequence was then cloned into the PDL2 plasmid vector and introduced into the protoplasts of the Δ*VP1G_08835-85* mutant, yielding the complemented strains Δ*VP1G_08835*-C-1 and Δ*VP1G_08835*-C-10. In parallel, the construct was introduced into wild-type *C. pyri* protoplasts, generating the overexpression strains *VP1G_08835*-OE-1 and *VP1G_08835*-OE-22.

Mycelial growth assays showed that deletion of *VP1G_08835* impaired vegetative growth of *C. pyri*. After 3 d of incubation, the mean colony diameters of WT, Δ*VP1G_08835*-85, and Δ*VP1G_08835*-107 were 66.94, 60.61, and 60.60 mm, respectively, representing reductions of 9.45% and 9.47% compared with WT. The complemented strain Δ*VP1G_08835*-C partially restored the growth phenotype (Figure 6C). Pathogenicity assays on detached twigs further showed that deletion of *VP1G_08835* reduced virulence, with lesion lengths reduced by 26.62% compared with WT, whereas complementation restored the WT phenotype (Figure 6D). These results indicate that *VP1G_08835* contributes to fungal growth and virulence. However, because deletion of *VP1G_08835* also affected vegetative growth in vitro, the reduced lesion development may partly result from impaired fungal fitness, and the present results cannot completely distinguish direct infection-related functions from indirect effects associated with reduced growth.

## 4. Discussion and Conclusions

In this study, the GH28 gene family of the pear canker pathogen *C. pyri* was systematically identified and analyzed at the sequence, structural, evolutionary, and functional levels. Most of the 15 GH28 proteins in *C. pyri* were predicted to be acidic, hydrophilic, and extracellularly localized, and they generally retained conserved motifs associated with the GH28 catalytic domain. These features are consistent with the established roles of fungal GH28 enzymes in extracellular pectin degradation, suggesting that this family may promote pathogen colonization and lesion expansion by disrupting the host cell wall [8,10,38,39]. Meanwhile, marked differences were observed among family members in protein length, motif composition, exon–intron organization, genomic distribution, and promoter architecture, indicating that individual GH28 proteins may have undergone functional diversification in substrate preference, catalytic efficiency, temporal expression, and interactions with host-derived inhibitors.

Three-dimensional structural comparison further revealed both conservation and divergence among the GH28 family members. The Phyre2-predicted models of all 15 proteins could be partially superimposed onto the experimentally determined fungal endo-polygalacturonase TlPGA structure, 6KVE-A [24,25]. Seven proteins showed relatively extensive structural similarity, with Cα-RMSD values ranging from 0.416 to 0.637 Å and 6KVE-A coverage ranging from 72.3% to 87.3%. The combination of low RMSD values and high structural coverage supports the conclusion that these proteins largely retain the characteristic GH28 fold [3,25]. Although the remaining members also showed relatively low RMSD values within the aligned regions, their overall structural coverage was more limited, suggesting greater variation in terminal extensions, inserted segments, or surface loops. These variable regions may affect substrate accessibility, protein stability, secretion efficiency, or interactions with host proteins, thereby providing a structural basis for functional diversification within the family.

*VP1G_08835* showed the strongest and most sustained induction during infection of pear twigs, reaching an expression peak of approximately 2.77-fold at 6 dpi. Its structural superposition with 6KVE-A yielded a Cα-RMSD of 0.912 Å, with target-model and 6KVE-A coverage values of 41.9% and 48.1%, respectively, indicating that its similarity to the reference structure was mainly confined to a partial GH28 structural region. Deletion of *VP1G_08835* impaired both vegetative growth and pathogenicity of *C. pyri*. The deletion mutants exhibited reduced colony growth and produced lesions on detached pear twigs that were 26.62% shorter than those caused by the wild-type strain, whereas complementation restored the corresponding phenotypes. These genetic results indicate that *VP1G_08835* contributes to normal fungal growth and full virulence. Together with its predicted extracellular localization and complete GH28 domain, these findings suggest that the encoded protein may participate in the modification of pectin or other host cell wall components in the extracellular environment [3,7,9,40]. The simultaneous effects of gene deletion on colony growth and lesion expansion further suggest that VP1G_08835 may contribute not only to host invasion but also to the broader physiological adaptation required for successful infection, thereby linking fungal fitness with host colonization capacity. Although deletion of *VP1G_08835* reduced lesion development, the mutant also exhibited impaired vegetative growth in vitro. Therefore, the reduced virulence phenotype may reflect both a direct contribution of VP1G_08835 to host infection and an indirect consequence of reduced fungal fitness. Further functional analyses, such as enzymatic characterization and host-interaction assays, are required to clarify its specific role during infection.

The differences in structural conservation and infection-induced expression among the 15 GH28 members suggest that this family is unlikely to consist of fully redundant pectin-degrading enzymes. Instead, its members may function at different stages of infection or perform distinct biochemical roles. VP1G_08835 represents an infection-associated member required for the full pathogenic performance of *C. pyri*, whereas other proteins with higher structural coverage may retain more typical GH28 enzymatic features. Overall, this study establishes an initial link among structural diversification, transcriptional responses, and pathogenicity within the *C. pyri* GH28 family and demonstrates the important role of *VP1G_08835* in fungal growth and virulence. Further characterization of its enzymatic activity, substrate specificity, and host cell wall degradation products will help clarify its precise role in pear canker development and may provide a basis for identifying potential targets for disease control.

## Figures and Tables

**Table 1:** Structural information of *C. pyri* GH28 genes.

Sequence ID	Number of Amino Acids	Molecular Weight	Theoretical Isoelectric Point (pI)	Instability Index	Aliphatic Index	Grand Average of Hydropathicity	Subcellular Localization
*VP1G_08384*	414	41,974.22	4.32	27.20	78.26	0.021	Extracell
*VP1G_00702*	430	47,050.52	4.60	23.61	75.42	−0.062	Extracell
*VP1G_08835*	475	51,563.56	5.09	33.99	76.19	−0.156	Extracell
*VP1G_03209*	461	50,326.97	4.52	39.38	79.28	−0.216	Extracell
*VP1G_01856*	371	38,820.05	4.48	29.72	88.46	−0.083	Extracell
*VP1G_02270*	758	82,574.07	4.82	44.12	73.21	−0.596	Extracell
*VP1G_05880*	445	47,503.72	4.47	25.17	73.24	−0.048	Extracell
*VP1G_06282*	391	40,267.62	4.76	23.80	75.58	−0.132	Extracell
*VP1G_03723*	456	46,796.17	4.40	34.23	69.30	−0.103	Extracell
*VP1G_00675*	440	48,014.64	5.22	29.39	78.43	−0.275	Extracell
*VP1G_08494*	462	47,171.11	4.25	30.57	78.14	0.087	Extracell
*VP1G_05365*	397	40,875.91	4.50	28.97	67.83	−0.143	Extracell
*VP1G_03817*	383	39,581.31	4.11	27.71	75.64	−0.163	Extracell
*VP1G_03614*	394	40,422.68	4.18	31.94	82.87	0.003	Extracell
*VP1G_03071*	327	35,573.51	4.50	42.35	74.25	−0.238	Cytosol

Gene symbols are shown in italics.

**Table 2:** Structural comparison of Phyre2-predicted *C. pyri* GH28 protein models with the crystal structure of TlPGA (PDB ID: 6KVE, chain A).

Reference Structure	Target Structure	Post-Refinement CA-RMSD (Å)	Aligned Cα Atoms (*n*)	Cα Atoms in Target Model (*n*)	Cα Atoms in 6KVE-A (*n*)	Target-Model Coverage (%)	6KVE-A Coverage (%)
6KVE-A	*VP1G_00675*	1.267	189	389	339	48.6	55.8
6KVE-A	*VP1G_00702*	0.943	136	370	339	36.8	40.1
6KVE-A	*VP1G_01856*	0.424	284	348	339	81.6	83.8
6KVE-A	*VP1G_02270*	0.843	162	406	339	39.9	47.8
6KVE-A	*VP1G_03071*	1.056	156	282	339	55.3	46
6KVE-A	*VP1G_03209*	0.861	157	384	339	40.9	46.3
6KVE-A	*VP1G_03614*	0.420	296	345	339	85.8	87.3
6KVE-A	*VP1G_03723*	0.637	275	344	339	79.9	81.1
6KVE-A	*VP1G_03817*	0.423	284	333	339	85.3	83.8
6KVE-A	*VP1G_05365*	1.333	205	371	339	55.3	60.5
6KVE-A	*VP1G_05880*	1.207	209	424	339	49.3	61.7
6KVE-A	*VP1G_06282*	0.416	285	342	339	83.3	84.1
6KVE-A	*VP1G_08384*	0.607	245	341	339	71.8	72.3
6KVE-A	*VP1G_08494*	0.637	272	343	339	79.3	80.2
6KVE-A	*VP1G_08835*	0.912	163	389	339	41.9	48.1

Notes: Target-model coverage was calculated as the number of aligned Cα atoms divided by the total number of Cα atoms present in the corresponding Phyre2 final PDB model × 100. The number of Cα atoms in the target model refers to the modeled residues present in the final PDB file and may therefore differ from the full-length amino acid sequence reported in Table 1. The 6KVE-A coverage was calculated as the number of aligned Cα atoms divided by the 339 Cα atoms present in the reference structure × 100. Post-refinement Cα-RMSD values and aligned Cα-atom counts represent the final values obtained after structural superposition in PyMOL. Gene symbols are shown in italics.

## Data Availability

The data in this study are available on request from the corresponding author/first author.

## Supplementary Materials

The following supporting information can be downloaded at: https://www.mdpi.com/article/10.3390/jof12080569/s1. Figure S1: Structural superposition of Phyre2-predicted *Cytospora pyri* GH28 protein models with the fungal endo-polygalacturonase TlPGA (PDB ID: 6KVE, chain A); Figure S2: Construction strategy and PCR screening of the *VP1G_08835* deletion mutants; Figure S3: PCR verification of the *VP1G_08835*-complemented strains; Figure S4: Molecular verification and expression analysis of the *VP1G_08835* overexpression strains; Figure S5: Specificity validation of RT-qPCR primer pairs for 15 *C. pyri* GH28 genes; Figure S6: Amplification curves of the RT-qPCR assays used for the expression analysis of *C. pyri* GH28 genes; Figure S7: Melting-curve analysis of the RT-qPCR products used for the expression analysis of *C. pyri* GH28 genes; Table S1: All primers used in this study.
