# Supplementary material for: Systematic Identification of the GH28 Gene Family in the Pathogenic Fungus Cytospora pyri and Functional Verification of the Candidate Virulence Gene VP1G_08835
Source: J Fungi (Basel). 2026 Aug 1;12(8):569. doi: 10.3390/jof12080569 (PMC13514856; doi:10.3390/jof12080569)
Supplement: Supplementary file 1 [file jof-12-00569-s001.zip › Supplementary1.pdf]

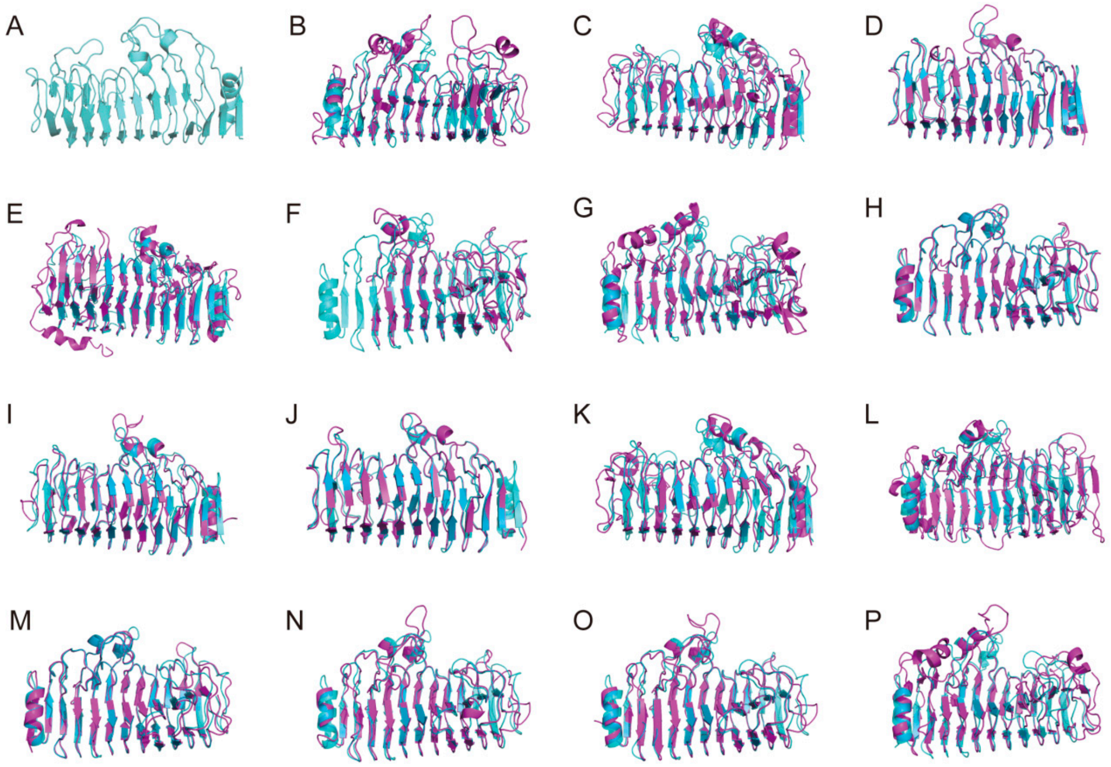

Supplementary Figure S1. Structural superposition of Phyre2-predicted *Cytospora pyri* GH28 protein models with the fungal endo-polygalacturonase TlPGA (PDB ID: 6KVE, chain A).

(A) Crystal structure of the reference protein 6KVE-A. (B–P) Structural superpositions of 6KVE-A with the predicted models of VP1G_00675 (B), VP1G_00702 (C), VP1G_01856 (D), VP1G_02270 (E), VP1G_03071 (F), VP1G_03209 (G), VP1G_03614 (H), VP1G_03723 (I), VP1G_03817 (J), VP1G_05365 (K), VP1G_05880 (L), VP1G_06282 (M), VP1G_08384 (N), VP1G_08494 (O), and VP1G_08835 (P). The reference structure 6KVE-A is shown in cyan, and the Phyre2-predicted target models are shown in magenta. Structural superposition was performed in PyMOL using Cα atoms. All structures are displayed in a comparable orientation to facilitate visualization of conserved and divergent regions.

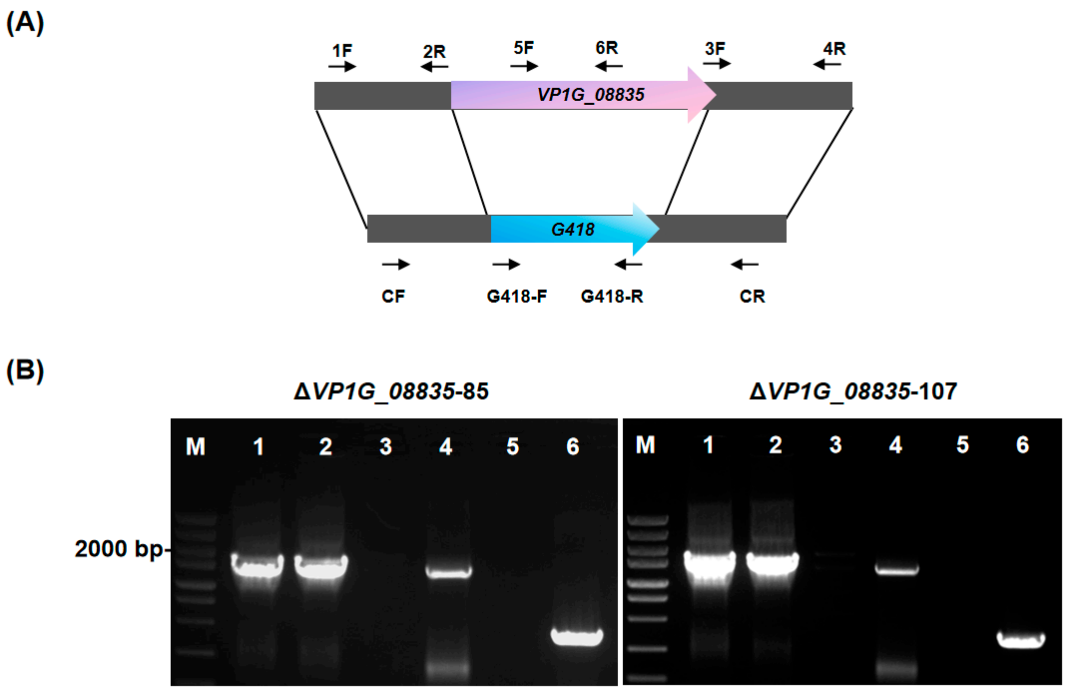

Supplementary Figure S2. Construction strategy and PCR screening of the *VP1G_08835* deletion mutants.

(A) Schematic representation of the replacement of the *VP1G_08835* coding region with the G418-resistance cassette. The positions and orientations of the primers used for construct preparation and mutant screening are indicated by arrows.

(B) PCR screening of two independently obtained deletion mutants, Δ*VP1G_08835*-85 and Δ*VP1G_08835*-107. M, DNA marker 5000 bp; lane 1, upstream flanking region amplified using primers 1F/2R; lane 2, downstream flanking region amplified using primers 3F/4R; lane 3, *VP1G_08835* coding region amplified using primers 5F/6R; lane 4, G418-resistance cassette amplified using primers G418-F/G418-R; lane 5, no-template control; lane 6, wild-type genomic DNA amplified using primers 5F/6R. The *VP1G_08835* coding-region product was not detected in either deletion mutant, whereas the G418-resistance cassette was detected. The expected *VP1G_08835* product was detected in the WT control. These results are consistent with deletion of *VP1G_08835* and the presence of the G418-resistance cassette in the selected transformants. Primer sequences are provided in Supplementary Table S1.

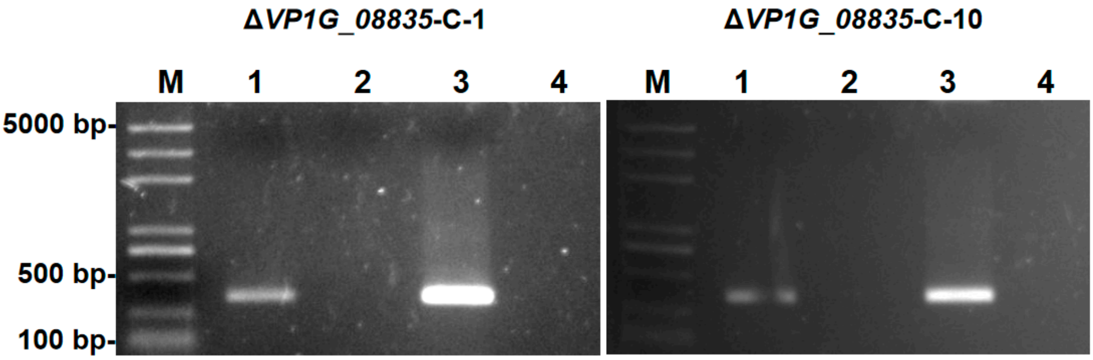

Supplementary Figure S3. PCR verification of the *VP1G_08835*-complemented strains.

Genomic PCR verification of two independently generated complemented strains, Δ*VP1G_08835*-C-1 and Δ*VP1G_08835*-C-10, using the primer pair 5F/6R. M, DNA marker 5000 bp; lane 1, genomic DNA of the wild-type strain, used as a positive control; lane 2, genomic DNA of the Δ*VP1G_08835* deletion mutant; lane 3, genomic DNA of the corresponding complemented strain; lane 4, no-template control. The expected *VP1G_08835* amplicon was detected in the wild-type and complemented strains but was absent from the deletion mutant and no-template control.

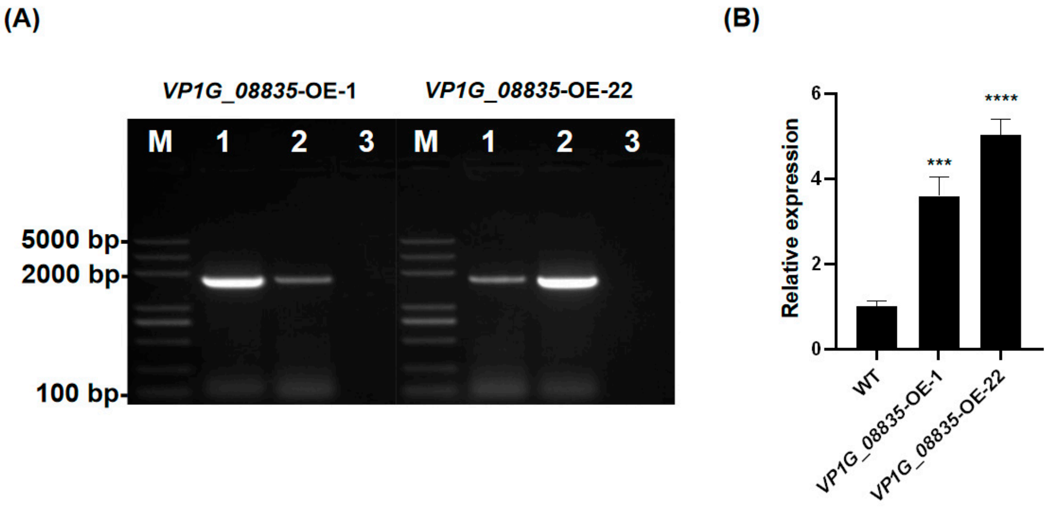

Supplementary Figure S4. Molecular verification and expression analysis of the *VP1G_08835* overexpression strains.

(A) PCR verification of two independently generated *VP1G_08835* overexpression strains, *VP1G_08835*-OE-1 and *VP1G_08835*-OE-22. M, DNA marker 5000 bp; lane 1, amplification of the endogenous *VP1G_08835* gene from the wild-type strain, used as a positive control; lane 2, amplification of the pDL2-derived overexpression construct from the corresponding overexpression strain using pDL2-specific primers; lane 3, amplification of wild-type genomic DNA using the same pDL2-specific primers, used as a negative control.

(B) Relative transcript levels of *VP1G_08835* in the wild-type strain and the independently generated overexpression strains *VP1G_08835*-OE-1 and *VP1G_08835*-OE-22, as determined by RT-qPCR. The transcript level in the wild-type strain was normalized to 1, and β-actin was used as the internal reference gene. Data are presented as the mean ± SD of three biological replicates. Statistical significance relative to the wild-type strain was determined using one-way ANOVA followed by Tukey's multiple-comparison test. ***, $P < 0.001$; ****, $P < 0.0001$.

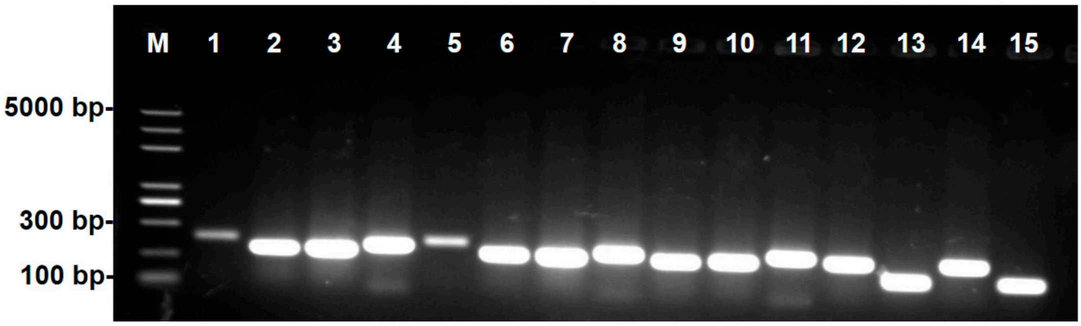

Supplementary Figure S5. Specificity validation of RT-qPCR primer pairs for 15 *C. pyri* GH28 genes.

PCR products generated using the RT-qPCR primer pairs were analyzed by agarose gel electrophoresis. M, DNA marker 5000 bp; lanes 1–15 correspond to *VP1G_08384*, *VP1G_00702*, *VP1G_08835*, *VP1G_03209*, *VP1G_01856*, *VP1G_02270*, *VP1G_05880*, *VP1G_06282*, *VP1G_03723*, *VP1G_00675*, *VP1G_08494*, *VP1G_05365*, *VP1G_03817*, *VP1G_03614*, and *VP1G_03071*, respectively. A single predominant amplicon was detected for each primer pair, supporting the specificity of the primers used for RT-qPCR analysis.

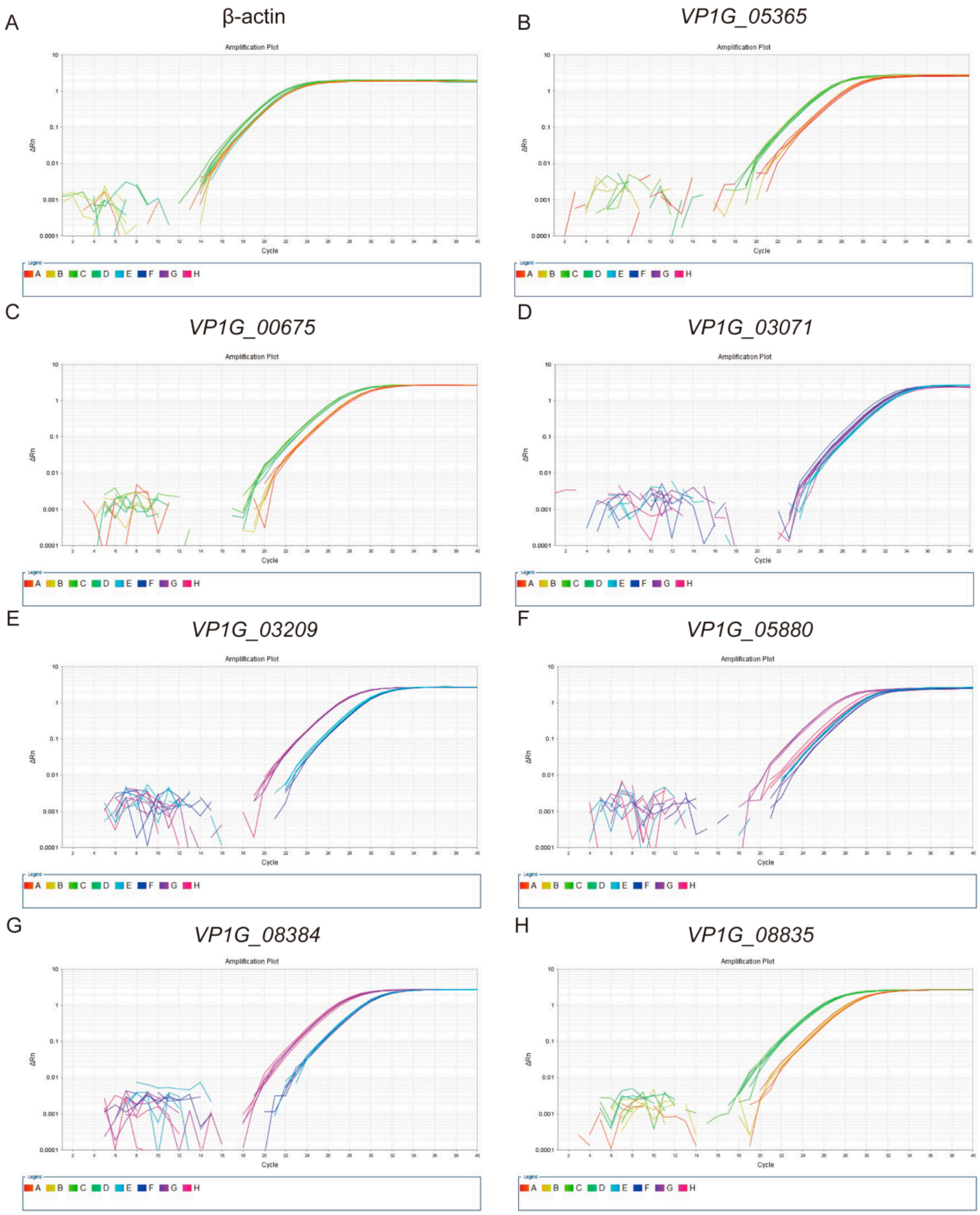

Supplementary Figure S6. Amplification curves of the RT-qPCR assays used for the expression analysis of *C. pyri* GH28 genes.

Amplification curves for (A) *β-actin*, (B) *VP1G_00675*, (C) *VP1G_03071*, (D) *VP1G_03209*, (E) *VP1G_05365*, (F) *VP1G_05880*, (G) *VP1G_08384*, and (H) *VP1G_08835*. The curves showed typical sigmoidal amplification profiles and generally consistent amplification among technical replicates.

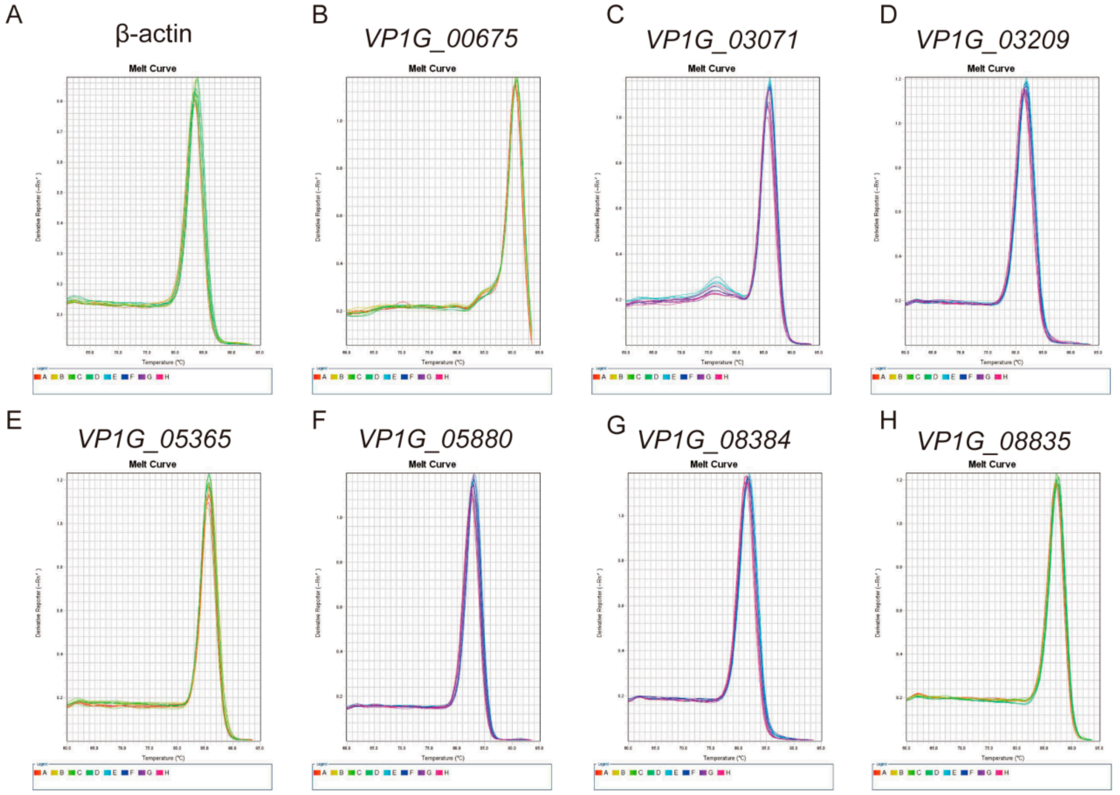

Supplementary Figure S7. Melting-curve analysis of the RT-qPCR products used for the expression analysis of *C. pyri* GH28 genes.

Melting curves for (A) *β-actin*, (B) *VP1G_00675*, (C) *VP1G_03071*, (D) *VP1G_03209*, (E) *VP1G_05365*, (F) *VP1G_05880*, (G) *VP1G_08384*, and (H) *VP1G_08835*. Most primer pairs produced a single dominant melting peak, supporting the specificity of the corresponding RT-qPCR amplification products.
